# Clinical and Diagnostic Features of West Nile Virus Neuroinvasive Disease in New York City

**DOI:** 10.3390/pathogens13050382

**Published:** 2024-05-03

**Authors:** Jackson A. Roberts, Carla Y. Kim, Amy Dean, Karen E. Kulas, Kirsten St. George, Hai E. Hoang, Kiran T. Thakur

**Affiliations:** 1 Program in Neuroinfectious Diseases, Department of Neurology, Columbia University Irving Medical Center, New York, NY 10032, USA; 2 Laboratory of Viral Diseases, Wadsworth Center, New York State Department of Health, Albany, NY 12237, USA; 3 Diagnostic Immunology, Wadsworth Center, New York State Department of Health, Albany, NY 12237, USA; 4 Department of Biomedical Science, University at Albany, SUNY, Albany, NY 12222, USA; 5 Department of Neurology, Weill Cornell Medical Center, New York, NY 10065, USA

**Keywords:** neuroinfectious disease, West Nile virus, encephalitis, neuroinvasive infections, meningitis, arboviral disease

## Abstract

West Nile virus (WNV) neuroinvasive disease (WNND) occurs in approximately 1 percent of WNV-infected patients and typically presents as encephalitis, meningitis, or acute flaccid paralysis (AFP). WNND remains a difficult inpatient diagnosis, creating significant challenges for prognostication and therapy selection. We characterized the clinical and diagnostic features of WNND cases at two major academic medical centers in New York City in routine clinical practice. We retrospectively reviewed the charts of thirty-six patients with WNND, including twenty-six encephalitis, four meningitis, and six AFP cases. The most common presenting symptoms were fever (86.1%) and gastrointestinal symptoms (38.9%) in addition to altered mental status (72.2%), lethargy (63.9%), gait disturbances (46.2%), and headache (44.4%). Fourteen (48.3%) patients displayed acute magnetic resonance imaging (MRI) findings, particularly T2 hyperintensities in the bilateral thalami, brainstem, and deep white matter. New York State Department of Health WNV CSF IgM testing was utilized for diagnosis in 58.3% of patients; however, just 38.1% had the result by discharge, compared to 85.6% of those who underwent serum IgM testing. The median length of stay was 13.5 days, 38.9% were intubated, and three patients (8.9%) died during acute hospitalization. Our findings underscore the morbidity, mortality, and diagnostic challenges of WNND, suggesting the potential utility of serum IgM testing in combination with confirmatory CSF testing to expedite diagnosis in the acute setting.

## 1. Introduction

West Nile virus (WNV) is a Flavivirus, primarily transmitted by *Culex* mosquitos [1], that is capable of causing severe neurologic disease in humans [2]. WNV has become endemic in the United States since its initial outbreak in New York City in 1999 [3,4], followed by a 2002 epidemic impacting 39 states [5]. While the initial strains circulating globally predominately caused subclinical or mild symptoms, the novel strain (lineage 1) that emerged in the 1990s and established reservoirs in the United States displays a greater propensity for neurological infections [6,7]. As of 2022, 28,684 cases of West Nile neuroinvasive disease (WNND) have been reported to the Centers for Disease Control (CDC) [8], and WNV now represents the most common cause of arboviral disease and the leading cause of arboviral encephalitis in the United States [9].

In most immunocompetent individuals, WNV infection is asymptomatic with less than 1% of individuals developing WNND [10]. The risk of developing WNND and experiencing severe disability or death increases with age, immunocompromised state, and medical comorbidities including chronic renal disease, hypertension, diabetes, and a history of alcohol use disorder [11,12,13]. WNND typically manifests as encephalitis, meningitis, or acute flaccid paralysis (AFP), most often a poliomyelitis-like syndrome with anterior horn cell involvement, but it may also resemble Guillain–Barré syndrome (GBS) with peripheral demyelination, in isolation or in combination [14,15]. Case fatality for WNND is approximately 9 percent and is highest in those with encephalitis or AFP, particularly in those older than 70 years [16].

Despite this significant burden of WNND, diagnosis of infection remains challenging and relies on a high index of clinical suspicion in combination with an understanding of the epidemiological context. For instance, even during a known outbreak of WNV in Arizona, just 40% of patients with clinically compatible meningitis or encephalitis were tested for WNV [17]. Delays in diagnosis present a serious challenge to developing and evaluating treatments for WNND, for which there exists a very limited evidence base, as prompt administration of therapies in acute infection is likely to yield the greatest clinical effect. In addition, the majority of studies examining the clinical features of WNND have utilized active surveillance during major outbreaks in the early 2000s, while more recent reports have primarily focused on immunocompromised subpopulations of patients, particularly solid organ transplant recipients [18,19,20,21].

A description of the clinical and diagnostic features of WNND in a non-outbreak population is therefore necessary to characterize the presentation and evaluation of such patients in routine clinical practice. As a result, we undertook a retrospective review at two large academic medical centers and affiliated sites to perform a robust clinical characterization of WNND cases, particularly considering diagnostic features that may support the earlier identification and management of WNND cases in non-outbreak situations without active public health surveillance.

## 2. Methods

### 2.1. Study Population

We retrospectively reviewed the medical records of patients aged 18 years and older with probable or definitive WNND between January 1, 2010 and November 30, 2023 at Columbia University Irving Medical Center (CUMC) and Weill Cornell Medical Center (WCMC) and their local affiliate hospital sites. Participants were identified by laboratory records or by ICD-10 codes followed by laboratory record confirmation. Diagnosis of WNND was established by detection of WNV anti-IgM antibodies in cerebrospinal fluid (CSF) or serum by enzyme-linked immunoassay (ELISA) with compatible features of neurologic illness (fever, headache, new focal neurological or cognitive deficits) in accordance with standard case definitions [4]. Laboratory testing was performed by both the New York State Department of Health (NYSDOH) Wadsworth Center and commercial laboratory testing (Quest Diagnostics, Secaucus, NJ; ARUP Laboratories, Salt Lake City, UT). Institutional review board (IRB) approval and a waiver of informed consent were obtained from the participating universities’ institutional review boards.

### 2.2. Case Definitions

Participants were grouped into categories of encephalitis, meningitis, and acute flaccid paralysis on the basis of clinical features of disease. The case definitions utilized, as previously defined [4], were (1) encephalitis: features of encephalopathy (depressed or altered mental status, lethargy, or personality change) with ≥2 of (a) fever (≥38 °C) or hypothermia (≤35 °C), (b) CSF pleocytosis (≥5 cells/mm^3^), (c) consistent neuroimaging findings, (d) presence of focal neurologic deficits, (e) meningismus, (f) electroencephalography (EEG) findings consistent with encephalitis, or (g) seizures; (2) meningitis: evidence of meningeal inflammation (nuchal rigidity, Kernig or Brudzinski sign, or photo- or phonophobia with ≥1 of (a) fever (≥38 °C) or hypothermia (≤35 °C), (b) CSF pleocytosis (≥5 cells/mm^3^), or (c) neuroimaging findings of acute meningeal inflammation; (3) acute flaccid paralysis (AFP): acute onset limb weakness with progression over 48 h with ≥2 of (a) asymmetric weakness, (b) areflexia or hyporeflexia of the affected limb(s), (c) absence of pain, paresthesia, or numbness in the affected limb(s), (d) CSF pleocytosis (≥5 cells/mm^3^) and elevated CSF protein (≥45 mg/dL), (e) electrodiagnostic testing suggestive of anterior horn cell involvement, or (f) magnetic resonance imaging (MRI) with abnormal signals in the spinal gray matter. AFP as a category includes patients with acute flaccid myelitis (AFM), referring to paralytic limb weakness with MRI abnormalities in the gray matter, as well as GBS. Individuals with features of meningitis or acute flaccid paralysis who satisfied the case definition for encephalitis were classified as encephalitis cases.

### 2.3. Outcomes

Data were obtained exclusively via retrospective chart review. In determining the occurrence of systemic and neurologic symptoms, symptoms were considered absent if not mentioned in the initial clinical history or consultant notes. CSF profile data were recorded from the first lumbar puncture (LP), whether performed at an outside hospital or on admission to local institutions in the study. Similarly, the first MRI with a full report available was utilized for consideration of radiographic findings. All MRIs were reviewed by a board-certified neuroradiologist. Electromyography (EMG) results were obtained by review of the full neurophysiologist’s report and impressions. EEG reports were reviewed to determine the presence of electrographic seizures. Glasgow Coma Scale (GCS) and modified Rankin Scale (mRS) were tabulated on admission and discharge on the basis of documented physical exam findings. The development of systemic complications during admission was determined by specific mention in clinical progress notes and confirmed by review of laboratory records as appropriate (i.e., syndrome of inappropriate antidiuretic hormone secretion (SIADH) or thrombocytopenia (<150,000 platelets/μL)).

### 2.4. Statistical Analysis

Data were summarized using descriptive statistics, including mean and standard deviation or median and range for continuous variables and counts and frequency for categorical variables. Analyses were performed using R Studio version 1.3.1073 (Posit, Boston, MA, USA).

## 3. Results

### 3.1. Patient Characteristics

A total of 36 patients with probable or definitive WNND were identified for inclusion in the study between September 2010 and August 2023. Patients were on average 61.4 years old (range: 33.9–87.2), 26 patients (72.2%) were male, and 20 (55.6%) patients self-identified as non-Hispanic white (Table 1). Encephalitis was the most common discharge diagnosis, occurring in twenty-six patients, while six patients were classified as AFP, and four as having meningitis. Two patients with encephalitis also displayed features of AFP. Medical comorbidities included hypertension in fourteen patients (38.9%), chronic kidney disease in five (13.9%), and coronary artery disease in eight (22.2%). Seventeen patients (47.2%) had an immunocompromising condition, including type II diabetes in eight (22.2%), history of transplant in five (13.9%), and prior or current cancer in five (13.9%). Transplant recipients included two patients with a liver transplant, two with a renal transplant, and one with a bilateral lung transplant. Patients with a medical history of cancer included two patients with prostate cancer, one with small cell lung cancer, one with renal cell carcinoma, and one with chronic lymphocytic leukemia. Thirteen patients (36.1%) reported current or former tobacco use, and sixteen (44.4%) reported current alcohol use.

### 3.2. Presenting Features

**Figure 1:**
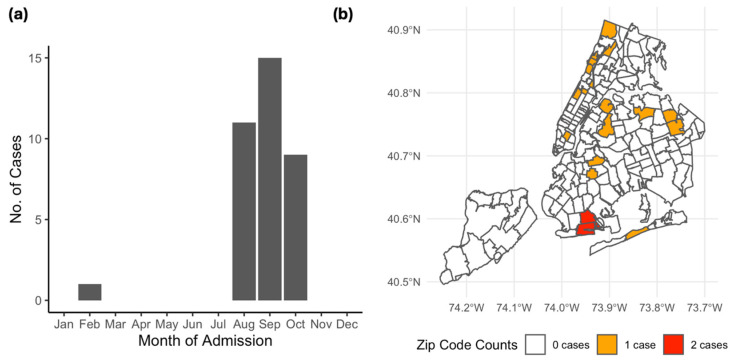
(**a**) Bar plot demonstrating frequency of month of admission among WNND patients. Bars represent the absolute count of patients presenting in each month. (**b**) Distribution of cases within New York City. Grey lines indicate the outlines of zip codes within New York City, with shading indicating the number of patients with home addresses in each zip code. Not shown are 3 zip codes in New Jersey (07010, 07701, 079101) with one case and 1 zip code in Yonkers with 2 patients (10704).

All patients except one, who had a subacute onset of symptoms and presented in February, presented between August and October (Figure 1). The patient presenting in February, who was immunosuppressed following a liver transplantation, had experienced the onset of leg weakness and dizziness at the end of August and had an inflammatory CSF profile with lymphocytic pleocytosis but had received no WNV antigen testing at that time. She experienced a progression of lower extremity weakness and developed an altered mental status, prompting admission and evaluation which yielded a diagnosis of WNV AFP. In terms of clinical history, eight (22.2%) patients recalled a mosquito bite in the month preceding presentation, eight (22.2%) had recently traveled to a wooded area, five (13.9%) were employed primarily outdoors, and four (11.1%) reported sick contacts at home (Table 2). Of the patients with a travel history, six had recently traveled outside of the greater New York City metropolitan area. Sixteen patients were transferred from an outside hospital, including twelve (46.2%) patients with encephalitis, one (25.0%) patient with meningitis, and one (25.0%) patient with AFP.

The most common systemic symptoms at the time of presentation were fever (n = 31; 86.1%), nausea and/or vomiting (n = 14; 38.9%), diarrhea (n = 12; 33.3%), shortness of breath (n = 7; 19.4%), and coughing (n = 6; 16.7%) (Table 2). The median length from onset of the first systemic symptom to hospital presentation was 3 days (interquartile range: 4.25; range: 0–31). Among patients with encephalitis (n = 26), the most common neurologic symptoms at the time of presentation were altered mental status (n = 22; 84.6%), lethargy (n = 19; 73.1%), tremor (n = 15; 57.7%), generalized weakness (n = 13; 50.0%), headache (n = 9; 34.6%), and gait disturbances (n = 8; 30.8%). For patients with meningitis (n = 4), neurologic symptoms at presentation included headache (n = 4; 100%), dizziness or vertigo (n = 3; 75.0%), neck stiffness (n = 2; 50.0%), and lethargy (n = 2; 50.0%). For those with AFP (n = 6), the most common neurologic symptoms included focal weakness (n = 6; 100%), headache (n = 3; 50.0%), altered mental status (n = 3; 50.0%), gait disturbances (n = 2; 33.3%), and lethargy (n = 2; 33.3%). The median time from onset of first neurologic symptoms to presentation was 4 days (interquartile range: 5.5; range: 0–172). On admission, the median Glasgow Coma Scale was 13 (range: 3–15) for patients with encephalitis, 15 (range: 15–15) for patients with meningitis, and 15 (range: 7–15) for patients with AFP. The median admission modified Rankin Scale was four (range: 1–5) for patients with encephalitis, two (range: 2–3) for patients with meningitis, and four (range: 3–5) for patients with.

### 3.3. Laboratory, Neuroimaging, and Neurophysiological Evaluation

Thirty-four (94.4%) patients had CSF profiles that were available for review (Table 3). Across all of the WNND categories, CSF studies from initial lumbar punctures revealed pleocytosis (median leukocyte count: 49.5 cells/mm^3^, range: 1–795), elevated protein (median: 97.5 mg/dL, range: 37–830), and normal glucose levels (median: 65 mg/dL, range: 43–93). The majority of the CSF profiles displayed a lymphocytic predominance (n = 25; 73.5% of those with LP), though four patients (11.8% of those with LP) displayed a neutrophilic predominance. Lumbar puncture was performed a median of 3 days following presentation (range: 0–27 days).

**Figure 2:**
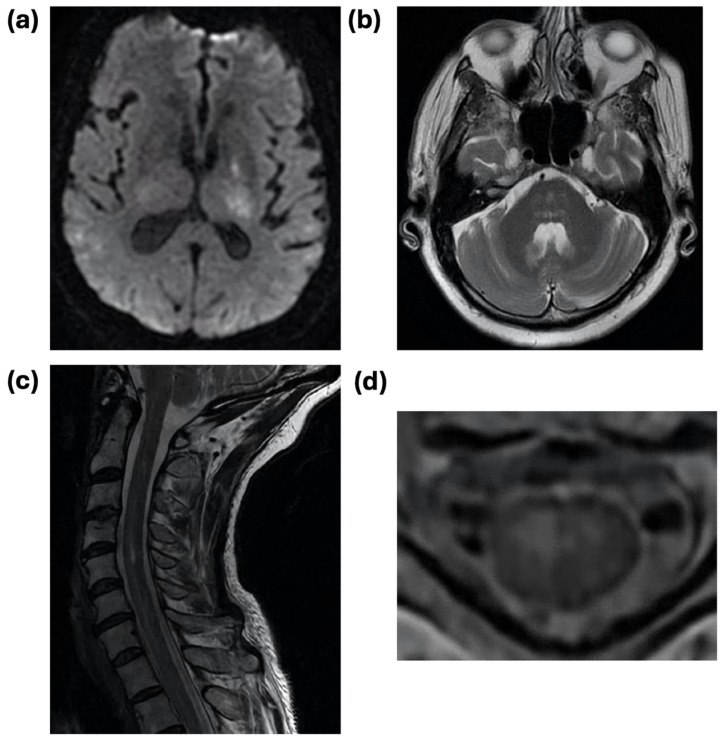
Representative radiographic features of WNV encephalitis and acute flaccid paralysis. (**a**) Diffusion-weighted imaging (DWI) axial MRI sequence demonstrating areas of restricted diffusion in the bilateral thalami with associated T2/FLAIR signal in an 83-year-old man with fatal WNV encephalitis who presented with dizziness, slurred speech, and falls. (**b**) T2/FLAIR axial MRI sequence demonstrating patchy bilateral signal in the pons in a 69-year-old man presenting with altered mental status and diagnosed with WNV encephalitis. (**c**) Sagittal T2 MRI sequence demonstrating hyperintensity of the cervical spinal cord extending from C3-C4 to the thoracic cord in a 57-year-old woman who presented with greater right than left proximal weakness progressing to respiratory failure, diagnosed with WNV acute flaccid paralysis. (**d**) Axial T2 MRI sequence of the patient in (**c**) demonstrating right greater than left cord hyperintensity, particularly of the ventral cord.

A brain MRI was performed on the majority of patients (n = 29; 80.6%). No patients with meningitis had acute MRI findings. For patients with encephalitis, the MRI displayed abnormal findings in 54.5% (n = 12) of those with imaging, including two with acute ischemic findings. Findings that were attributable to WNV infection included T2/FLAIR hyperintensities, varying with diffusion restriction, in the subcortical white matter, bilateral thalami, cerebral peduncles, internal capsule, pons, and cerebellum without contrast enhancement (Figure 2). Acute brain MRI findings in those with AFP who had both brain and spine imaging included diffuse pachymeningeal enhancement and right frontal T2/FLAIR hyperintensities in the right frontal subcortical and the deep white matter. A spinal MRI was performed on eight patients with encephalitis and five with AFP. Two (25.0%) patients with encephalitis (both of whom had additional clinical features that were compatible with AFP) had acute spinal MRI findings, including cauda equina enhancement in one patient and T2/FLAIR hyperintensity of the cervical gray matter without corresponding enhancement or edema and enhancement of the cauda equina nerve roots in another. Spinal MRI was abnormal in all of the AFP patients with imaging. Findings included longitudinally extensive (i.e., ≥3 spinal levels) T2/FLAIR hyperintensities of the cervical and thoracic cord without contrast enhancement, multifocal and asymmetric linear cervical cord hyperintensities, hyperintensity of the distal conus medullaris, and enhancement and thickening of the cauda equina nerve roots.

EMG or nerve conduction studies (NCS) were performed on six (16.7%) patients, two with encephalitis and AFP and four patients with AFP. The findings indicated a predominately motor axonopathy in four patients and a demyelinating polyneuropathy in two patients. Two patients underwent a brain biopsy, both due to the persistence of severe symptoms requiring prolonged ICU stays without a leading diagnosis. In one patient, a biopsy of a T2-hyperintense left frontal lobe lesion revealed mild-to-moderate astrogliosis and microgliosis with an inflammatory T-cell infiltrate. A biopsy of a T2-hyperintense right temporal lobe lesion in the second patient revealed prominent gliosis with marked astrogliosis and microgliosis in addition to a rich perivascular B-cell infiltrate, perivascular macrophages, and scattered and perivascular T-cell infiltrate.

### 3.4. West Nile Virus Diagnostic Testing

CSF testing for WNV anti-IgM antibodies was carried out and was positive for 24 (66.7%) cases, while the remainder had positive serological tests (Table 4). Eight patients had both serum and CSF anti-IgM positivity. The majority of CSF testing was performed by the Wadsworth Center, NYSDOH. The median time from collection to result for NYSDOH CSF testing was 16 days (range: 7–33 days), and the result was available by time of discharge for only eight patients (38.1%). CSF testing performed at commercial laboratories had a median time from collection to result of 8.5 days (range: 5–14 days) and was present by discharge in all cases. Serologic testing, which was performed for 21 (58.3%) patients, exclusively at commercial laboratories, had a median time from collection to result of 4 days (range: 2–16 days), was positive in 90.5% (n = 19) of cases, and the result was present at discharge for 85.7% (n = 18) of the patients, including 92.3% of encephalitis and 100% of AFP patients with serologic testing. Urine PCRs or antigen tests were not performed for any patients in the study. CSF WNV polymerase-chain reaction (PCR) was performed for nineteen patients but was positive for only one patient, who additionally had positive serum and whole blood WNV PCR positivity.

### 3.5. Hospital Course and Outcomes

The median length of hospitalization for the overall sample was 13.5 days (range: 3–60 days): 13 days (range: 3–60 days) for encephalitis patients, 4.5 days (range: 3–5 days) for meningitis patients, and 22.5 days (range: 7–47 days) for AFP patients (Table 5). Eighteen (50%) patients were admitted to the intensive care unit (ICU), including 41.7% (n = 15) of patients with encephalitis and 50.0% (n = 3) of patients with AFP, with a median ICU length of stay of 9.5 days (range: 1–51). Fourteen (38.9%) of the patients who were extubated by discharge required intubation and mechanical ventilation for a median length of 8 days (range: 6–63 days). Five encephalitis and one AFP patient remained intubated and required a tracheostomy prior to discharge. Only one patient with encephalitis had EEG-recorded seizures during admission. Overall, seven (19.4%) patients received a specific treatment for WNV infection, including four (66.7%) patients with AFP and three (11.5%) patients with encephalitis. A five-day course of intravenous immunoglobulin (IVIG) was completed in six patients, while one patient received dexamethasone for increased intracranial pressure. Systemic complications included urinary retention in eight patients (22.2%), aspiration pneumonia in seven patients (19.4%), thrombocytopenia in fourteen patients (38.9%), and SIADH in four patients (11.1%). Three patients (8.3%) in the study died during acute hospitalization, all of whom were diagnosed with encephalitis. All patients with meningitis were discharged home, compared to 23.1% (n = 6) of the patients with encephalitis and 16.7% (n = 1) of the patients with AFP.

## 4. Discussion

In this large, consecutive case series, we have sought to comprehensively characterize the presenting and diagnostic features of WNND, as well as the hospital course and clinical outcomes of these patients that were identified during routine clinical practice. Overall, the proportion of WNND patients developing encephalitis (72.2%) parallels that in prior work that found that 50–70% of hospitalized WNND patients develop encephalitis [4,22,23], and our sample contains a relatively low frequency of WNV meningitis cases (11.1%). Similar to what has been reported in prior work, patients most often presented with a fever and altered mental status, and gastrointestinal symptoms were a common finding, occurring in nearly 40% of patients [4,23,24,25,26,27,28,29,30,31,32]. In addition to an altered mental status, we found a tremor to be a common finding in WNV encephalitis patients, occurring in 50% of patients by the time of presentation, which parallels prior studies that found a frequency of tremor ranging from approximately one-fourth of WNV encephalitis patients to more than 90% [4,23,25,26]. In general, movement disorders are common in WNND, predominantly a coarse bilateral tremor of the upper extremities but also including opsoclonus-myoclonus and parkinsonism [33]. While other causes of infectious encephalitis display a similar clinical presentation, patients with a gastrointestinal or flulike illness followed by an altered mental status with a tremor or focal weakness presenting in late summer through early fall should prompt diagnostic testing for WNV.

Our sample was overwhelmingly male (72.2%), a predominance which has been found in some studies [34,35,36,37], though the reasons for an increased male risk for WNND remain unclear. While clinical history remains essential for diagnosis, a relatively low proportion of patients reported exposures, such as mosquito bites or significant outdoor activity, with an obvious connection to arboviral disease. Patient residences were distributed widely throughout New York City and its surrounding boroughs, reflecting the widespread distribution of the urban-adapted *Culex* mosquitos in the area, which may render classical exposures less useful in the urban context [38]. However, the patients nearly exclusively presented between August and October, reflecting the amplification of mosquito WNV infection rates in the late summer and early fall [39].

From a diagnostic perspective, patients almost invariably presented with CSF leukocytosis and elevated protein. Compared to prior studies that found a high frequency of neutrophilic predominance in WNND, we found only four patients (11.1%; data not shown) with greater than 50% neutrophils in CSF [29,40,41,42]. Though this study was underpowered to compare CSF profiles across disease categories, encephalitis patients interestingly had higher levels of CSF protein and leukocytosis, which is consistent with prior work [40]. Supportive MRI findings were present for the majority of encephalitis and all AFP patients, reflecting the involvement of the deep brain structures, particularly the bilateral thalami, brainstem, basal ganglia, and cerebral peduncles [23,43,44]. These findings are nonspecific for WNND but should raise clinical suspicion in a patient with compatible clinical and epidemiological factors. Electrophysiological testing further underscored a predominance of a poliomyelitis-like syndrome involving the motor axons of the anterior horn in those with focal weakness but could also be used to identify patients with WNV-induced GBS. An EEG may be pursued in these patients but is unlikely to reveal a characteristic pattern in WNND; seizures are uncommon in WNND patients as both a presenting feature and a complication of the disease [45].

Compared to most clinical studies of WNND, the patients included in this study represent a more clinically severe patient population, reflective of the academic medical center setting from which the patients were recruited and the high proportion of outside hospital transfers in our sample. One-half of our patients were admitted to the ICU, and approximately 40% of patients required mechanical ventilation during acute hospitalization, of whom only one-half were extubated by discharge. Despite this, the overall mortality in this sample (8.3%) was comparable to prior studies, and the mortality among ICU cases was 12.5%, compared to 25% in one prior study of ICU WNND cases [46]. As in prior work, there was infrequent use of targeted therapeutics for WNND; IVIG was utilized exclusively in those with features of acute flaccid paralysis. One small randomized control trial evaluating the efficacy of anti-WNV antibody-enriched immunoglobulin against standard IVIG and normal saline for patients with WNND found no difference in disability or death between treatment groups [47]. The higher proportion of AFP patients receiving IVIG may reflect provider experience with IVIG’s efficacy for GBS [48]. However, its efficacy has not been demonstrated for other causes of AFP and its use is not supported by guidelines for WNND at this time, though further studies are needed given the paucity of existing data. The mainstay of WNND treatment remains supportive care and targeted therapy for complications that develop. Evaluating the efficacy of treatments for WNND remains essential; however, recruitment for randomized clinical trials is stymied by the low number of WNND patients at any one institution and is further complicated by the significant latency to diagnosis of WNV infection.

To this end, we aimed to evaluate the diagnostic features of WNND in this study. We found that a substantial number of patients were not diagnosed with WNV infection by the time of discharge, particularly those with only CSF testing at New York State’s public health laboratory. Antibody testing remains essential for WNND diagnosis, as WNV infection typically causes only a low-level viremia that is undetectable by PCR [49]. Serum testing for anti-WNV IgM, however, was performed more rapidly and the results were available prior to discharge in most cases, including nearly 93% of encephalitis cases, warranting the consideration of this as an initial diagnostic test while awaiting confirmatory CSF testing. Previous studies have demonstrated that commercial serum WNV IgM testing has a sensitivity of 86–96% and a specificity approaching 100% [50,51] but is likely underutilized in the clinical setting. Though not evaluated in this study, others have explored the utility of PCR testing of different tissue matrices in WNV infection, particularly noting that urine and whole-blood PCRs may have the greatest sensitivity [52,53,54,55,56]. Given the ease of sampling, peripheral sampling for PCR may be considered for suspected cases but should not replace antigen testing in serum and CSF. Rapid serological testing may have a more essential role in WNND for early exclusion of other viral encephalitis etiologies with targeted treatments and more sensitive testing parameters, most notably herpes simplex virus (HSV) 1 and 2 and varicella zoster virus (VZV) infections [57]. PCR testing is also more sensitive for CNS infections perpetrated by enteroviruses and other arboviruses besides WNV, indicating its utility for supporting alternative diagnoses when WNND is suspected but unconfirmed [58].

Our study has several limitations. First, the retrospective nature of the study may limit the accuracy and depth of description of some clinical features of disease and precluded analysis of longitudinal follow-up data. In addition, as mentioned previously, the setting of the study at two major referral centers likely biased the sample towards more severe WNND cases warranting ICU admission, which may not be representative of the overall WNND population. The small sample size also prevented an analysis of differences between disease categories and factors associated with outcome or prognosis. In addition, we were unable to account for differences in blood and CSF sample processing or storage time at different laboratory facilities. In addition, plaque-reduction neutralization tests (PRNTs) were not performed for confirmation of WNV testing; however, the CDC diagnostic guidelines were still followed, including negative antigen testing for other endemic arboviral diseases. Lastly, this sample likely failed to capture a substantial number of patients at our institutions with WNND who did not undergo diagnostic testing during acute hospitalization and does not provide an accurate representation of the incidence of WNND in the region.

Taken together, we have sought to provide a robust characterization of the clinical, radiographic, laboratory, and diagnostic features of WNND at two major, urban academic medical centers. We have identified a substantial degree of morbidity due to WNND and potential areas for improvement in terms of diagnostic testing, as well as the need for further evaluation of the diagnostic and therapeutic modalities that can be used in WNND treatment.

## Figures and Tables

**Table 1:** Patient demographics.

	Total (n = 36)	Encephalitis (n = 26) *	Meningitis (n = 4)	Acute Flaccid Paralysis (n = 6)
Age ^#^	61.4 (13.1)	63.7 (13.8)	52.6 (12.8)	57.0 (6.0)
Male sex	26 (72.22%)	20 (76.9%)	3 (75.0%)	3 (50.0%)
Ethnicity				
Non-Hispanic White	20 (55.6%)	13 (50.0%)	2 (50.0%)	5 (83.3%)
Non-Hispanic Black	3 (8.3%)	3 (11.5%)	-	-
Hispanic	4 (11.1%)	3 (11.5%)	1 (25.0%)	-
Asian	3 (8.3%)	2 (7.7%)	1 (25.0%)	-
Other	2 (5.6%)	2 (7.7%)	-	-
Unknown	4 (11.1%)	3 (11.5%)	-	1 (16.7%)
Current or former tobacco use	13 (36.1%)	11 (30.6%)	1 (25.0%)	1 (16.7%)
Current alcohol use	16 (44.4%)	12 (46.2%)	3 (75.0%)	1 (16.7%)
Hypertension	14 (38.9%)	11 (42.3%)	1 (25.0%)	2 (33.3%)
Coronary artery disease	8 (22.2%)	8 (30.8%)	-	-
Chronic kidney disease	5 (13.9%)	5 (19.2%)	-	-
Any immunocompromising condition	17 (47.2%)	14 (53.8%)	1 (25.0%)	2 (33.3%)
Type II diabetes	8 (22.2%)	6 (23.1%)	1 (25.0%)	1 (16.7%)
History of transplant	5 (13.9%)	4 (15.4%)	-	1 (16.7%)
History of or current cancer	5 (13.9%)	5 (19.2%)	-	-

* Two additional patients with AFP; ^#^ Mean (standard deviation).

**Table 2:** Clinical history and symptoms at presentation.

	Total (n = 36)	Encephalitis (n = 26) *	Meningitis (n = 4)	Acute Flaccid Paralysis (n = 6)
Recalled mosquito bite(s)	8 (22.2%)	6 (23.1%)	2 (50.0%)	-
Recent travel to wooded area	8 (22.2%)	6 (23.1%)	2 (50.0%)	-
Predominately works outdoors	5 (13.9%)	3 (11.5%)	1 (25.0%)	1 (16.7%)
Other sick contacts	4 (11.1%)	4 (15.4%)	-	-
Transferred from outside hospital	16 (44.4%)	12 (46.2%)	1 (25.0%)	3 (50.0%)
***Systemic Symptoms***				
Fever	31 (86.1%)	23 (88.5%)	4 (100%)	4 (66.7%)
Nausea and/or vomiting	14 (38.9%)	11 (42.3%)	-	3 (50.0%)
Diarrhea	12 (33.3%)	11 (42.3%)	-	1 (16.7%)
Abdominal pain	5 (13.9%)	5 (19.2%)	-	-
Rash	3 (8.3%)	2 (7.7%)	-	1 (16.7%)
Cough	6 (16.7%)	5 (19.2%)	-	1 (16.7%)
Shortness of breath	7 (19.4%)	4 (15.4%)	1 (25.0%)	2 (33.3%)
***Neurologic Symptoms***				
Headache	16 (44.4%)	9 (34.6%)	4 (100%)	3 (50.0%)
Neck stiffness	7 (26.9%)	4 (15.4%)	2 (50.0%)	1 (16.7%)
Lethargy	23 (63.9%)	19 (73.1%)	2 (50.0%)	2 (33.3%)
Altered mental status	26 (72.2%)	22 (84.6%)	1 (25.0%)	3 (50.0%)
Seizures/convulsions	-	-	-	-
Generalized weakness	15 (41.7%)	13 (50.0%)	-	2 (33.3%)
Focal weakness	9 (25.0%)	3 (11.5%)	-	6 (100%)
Tremor	15 (41.7%)	15 (57.7%)	-	-
Gait disturbance	12 (46.2%)	8 (30.8%)	2 (50.0%)	2 (33.3%)
Dizziness or vertigo	10 (27.8%)	7 (26.9%)	3 (75.0%)	-

* Two additional patients with AFP.

**Table 3:** Laboratory, neuroimaging, and neurophysiological evaluation.

	Total (n = 36)	Encephalitis (n = 26)	Meningitis (n = 4)	Acute Flaccid Paralysis (n = 6)
CSF profile obtained	34 (94.4%)	24 (92.3%)	4 (100%)	6 (100%)
Leukocyte count (cells/mm^3^) ^#^	49.5 (1–795)	52.5 (6–795)	40 (15–280)	29 (1–234)
Protein (mg/dL)	97.5 (37–830)	100.5 (46–830)	85.5 (37–136)	81.5 (56–109)
Glucose (mg/dL)	65 (43–93)	66 (43–93)	64 (45–89)	64.5 (60–84)
Brain MRI performed	29 (80.6%)	22 (84.6%)	3 (75.0%)	4 (66.7%)
Any acute abnormality	14 (48.3%)	12 (54.6%)	0 (0.0%)	2 (50.0%)
Spine MRI performed	13 (36.1%)	8 (30.8%)	-	5 (83.3%)
Any acute abnormality	7 (53.9%)	2 (25.0%)	-	5 (100%)
EMG/NCS performed	6 (16.7%)	2 (7.7%) *	-	4 (66.7%)
Demyelinating polyneuropathy	2 (33.3%)	-	-	2 (50.0%)
Predominately motor axonopathy	4 (66.7%)	2 (100%)	-	2 (50.0%)

* Both with clinical features consistent with a diagnosis of AFP; ^#^ median (range).

**Table 4:** West Nile virus anti-IgM antibody testing.

	Total (n = 36)	Encephalitis (n = 26)	Meningitis (n = 4)	Acute Flaccid Paralysis (n = 6)
*NYSDOH CSF IgM sent*	21 (58.3%)	16 (61.5%)	-	5 (83.3%)
Time from collection to result, days ^#^	16 (7–33)	16.5 (7–33)	-	15 (12–18)
Positive	21 (100%)	16 (100%)	-	5 (100%)
Result present by discharge	8 (38.1%)	5 (31.3%)	-	3 (60.0%)
*Serum IgM sent*	21 (58.3%)	14 (53.9%)	4 (100%)	3 (50.0%)
Time from collection to result, days ^#^	4 (2–16)	5 (2–16)	4 (3–7)	4 (3–4)
Positive	19 (90.5%)	12 (85.7%)	4 (100%)	3 (100%)
Result present by discharge	18 (85.7%)	13 (92.9%)	2 (50.0%)	3 (100%)
*Other CSF IgM sent*	4	4	-	-
Time from collection to result, days ^#^	8.5 (5–14)	8.5 (5–14)	-	-
Positive	4 (100%)	4 (100%)	-	-
Result present by discharge	4 (100%)	4 (100%)	-	-

^#^ median (range).

**Table 5:** Hospital course and clinical outcomes.

	Total (n = 36)	Encephalitis (n = 26)	Meningitis (n = 4)	Acute Flaccid Paralysis (n = 6)
Admission GCS	14 (3–15)	13 (3–15)	15 (15–15)	15 (7–15)
Admission mRS	4 (1–5)	4 (1–5)	2 (2–3)	4 (3–5)
Admitted to ICU ^#^	18 (50.0%)	15 (41.7%)	-	3 (50.0%)
ICU length of stay, days	9.5 (1–51)	9 (1–51)	-	19 (8–32)
Intubation required *	14 (38.9%)	11 (42.3%)	-	3 (50.0%)
Length of mechanical ventilation, days ^$^	8 (6–63)	10.5 (6–63)	-	8 (8–8)
Extubated by discharge	7 (50.0%)	6 (54.6%)	-	1 (33.33%)
EEG-recorded seizures	1 (2.8%)	1 (3.85%)	-	-
Specific treatment given for WNND	7 (19.4%)	3 (11.5%)	-	4 (66.7%)
IVIG	6 (16.7%)	2 (7.7%)	-	4 (66.7%)
Dexamethasone	1 (2.8%)	1 (3.9%)	-	-
Urinary retention	8 (22.2%)	6 (23.1%)	-	2 (33.3%)
Aspiration pneumonia	7 (19.4%)	4 (15.4%)		3 (50.0%)
Thrombocytopenia	14 (38.9%)	10 (38.5%)	-	4 (66.7%)
SIADH	4 (11.1%)	4 (15.4%)	-	-
In-hospital mortality	3 (8.3%)	3 (11.5%)	-	-
Length of stay, days	13.5 (3–60)	14 (3–60)	4.5 (3–5)	22.5 (7–47)
Discharge GCS	15 (6–15)	15 (6–15)	15 (15–15)	15 (14–15)
Discharge mRS	4 (0–6)	4 (0–6)	1.5 (1–2)	4 (4–5)
Discharged to home	11 (30.6%)	6 (23.1%)	4 (100%)	1 (16.67%)

Continuous measures are given as median (range); ^#^ Includes admission to the ICU at study sites only, not outside hospital ICU admissions; * Includes 8 patients intubated at an outside hospital who remained intubated on admission; ^$^ If intubated at an outside hospital, length of ventilation refers only to the admission sites, as date of intubation was not available for all outside hospital records.

## Data Availability

The datasets presented in this article are not readily available because they contain protected patient information. Requests for access to the datasets can be directed to Dr. Kiran Thakur at the corresponding author’s email address.
